# Quantifying the Impact of Soil Moisture Sensor Measurements in Determining Green Stormwater Infrastructure Performance

**DOI:** 10.3390/s25010027

**Published:** 2024-12-24

**Authors:** Matina Shakya, Amanda Hess, Bridget M. Wadzuk, Robert G. Traver

**Affiliations:** 1 Department of Civil, Architectural and Environmental Engineering, Drexel University, 3141 Chestnut Street, Philadelphia, PA 19104, USA; 2 Department of Civil and Environmental Engineering, Villanova University, 800 Lancaster Avenue, Villanova, PA 19085, USA; amanda.hess@villanova.edu (A.H.); bridget.wadzuk@villanova.edu (B.M.W.); robert.traver@villanova.edu (R.G.T.)

**Keywords:** soil moisture calibration, dielectric properties, volumetric water content, uncertainty analysis, subsurface hydrology

## Abstract

The ability to track moisture content using soil moisture sensors in green stormwater infrastructure (GSI) systems allows us to understand the system’s water management capacity and recovery. Soil moisture sensors have been used to quantify infiltration and evapotranspiration in GSI practices both preceding, during, and following storm events. Although useful, soil-specific calibration is often needed for soil moisture sensors, as small measurement variations can result in misinterpretation of the water budget and associated GSI performance. The purpose of this research is to quantify the uncertainties that cause discrepancies between default (factory general) sensor soil moisture measurements versus calibrated sensor soil moisture measurements within a subsurface layer of GSI systems. The study uses time domain reflectometry soil moisture sensors based on the ambient soil’s dielectric properties under different soil setups in the laboratory and field. The default ‘loam’ calibration was compared to soil-specific (loamy sand) calibrations developed based on laboratory and GSI field data. The soil-specific calibration equations used a correlation between dielectric properties (real dielectric: *ε_r_*, and apparent dielectric: *K_a_*) and the volumetric water content from gravimetric samples. A paired *t*-test was conducted to understand any statistical significance within the datasets. Between laboratory and field calibrations, it was found that field calibration was preferred, as there was less variation in the factory general soil moisture reading compared to gravimetric soil moisture tests. Real dielectric permittivity (*ε_r_*) and apparent permittivity (*K_a_*) were explored as calibration options and were found to have very similar calibrations, with the largest differences at saturation. The *ε_r_* produced a 6% difference while the *K_a_* calibration produced a 3% difference in soil moisture measurement at saturation. *K_a_* was chosen over *ε_r_* as it provided an adequate representation of the soil and is more widely used in soil sensor technology. With the implemented field calibration, the average desaturation time of the GSI was faster by an hour, and the recovery time was quicker by a day. GSI recovery typically takes place within 1–4 days, such that an extension of a day in recovery could result in the conclusion that the system is underperforming, rather than it being the result of a limitation of the soil moisture sensors’ default calibrations.

## 1. Introduction

### 1.1. Motivation

Soil moisture is the key variable in understanding the hydrological processes within green stormwater infrastructure (GSI) systems, which play a vital role in managing urban stormwater and mitigating flooding. Soil moisture sensors can be used to track the movement of water through a GSI system’s soil media [1,2] to resolve the system’s water balance [3] during and after storm events. This understanding is crucial for optimizing system performance, improving resilience to extreme weather conditions, and identifying maintenance needs. Originally developed for agriculture operations, these sensors are used in stormwater research, although with different intended purposes than in agriculture, and therefore require different accuracy and precision [4,5] due to varying objectives such as quantifying the volume storage and recovery process.

Rainfall patterns influenced by climate change add an additional layer of complexity to GSI soil moisture dynamics [6]. The increasing frequency and intensity of extreme precipitation events, coupled with longer dry periods, can place additional stress on GSI systems. The spatial and temporal precision required for GSI systems often falls short, especially under changing climatic conditions that may exacerbate discrepancies in sensor measurements [7]. Researchers have examined their use [8,9,10,11,12] in a variety of GSI applications, such as bioretention and tree trenches. However, there is difficulty in interpreting observed data from these sensors in GSI, which inhibits their widespread use because the spatial and temporal precision required for GSI systems is not adequate [13].

Modern soil moisture sensors such as Hydraprobe II, Teros 12, etc., rely on the dielectric properties of soil to measure volumetric water content. However, ambient conditions and field installation complexities often introduce uncertainties in sensor readings, especially in settings like GSI, which have high porosity and diverse environmental conditions. While laboratory-based calibration provides a controlled approach to sensor validation, the variability in field conditions, such as the presence of salts or non-uniform soil textures, can lead to discrepancies between measured and actual soil moisture.

Given these challenges, this study seeks to address the gaps in soil moisture measurement accuracy for GSI applications. Specifically, it aims to quantify the uncertainties that cause discrepancies between default soil moisture measurements versus sensor-calibrated soil moisture to better understand the temporal evolution of GSI capacity. This research evaluates the impact of these discrepancies on the annual performance of GSI systems, focusing on subsurface indicators such as soil moisture recession, desaturation time, and soil moisture at saturation. The objective of this study is to provide actionable insights into sensor calibration and data interpretation for improving GSI design, monitoring, and maintenance under diverse environmental conditions.

### 1.2. Soil Moisture Sensor Background

There are several methods used to determine soil moisture content, ranging from classical, laboratory-based discrete sample techniques (e.g., thermo-gravimetric or calcium carbide) to modern technologies that measure a continuous soil moisture change (e.g., soil resistivity sensors, tensiometers, infrared moisture balance, or dielectric techniques; [14]). The thermo-gravimetric technique (TGM) is a standard reference for determining soil moisture content [14,15], where the wet soil sample is oven dried for 24 h at 110 °C, and the subsequent dry weight is recorded. It is a reliable, accurate, and cost-effective laboratory technique [8,14,16]. However, this method does not provide site-based continuous measurements over time. There are also in situ soil moisture sensors. where the observed reading is based on the dielectric properties of the soil, which use the principle of time domain reflectometry (TDR). Other in situ sensors, such as tensiometers, make observations based on soil moisture tension [17]. The TDR sensors that use the soil’s dielectric properties are preferred as they are considered one of the best available electronic techniques for determining water content; the measurement is based on pulse travel time to measure dielectric properties, which is paired with a calibration equation to yield a soil moisture reading [9].

Dielectric properties are expressed as the real component of dielectric permittivity (*ε_r_*), imaginary component of dielectric permittivity (*ε_i_*), and apparent permittivity (*K_a_*). *ε_r_* is associated with energy storage in the form of rotational or orientation polarization, which indicates the soil water content. Any changes in *ε_r_* are related to changes in water content. *ε_i_* measurements provide an opportunity to account for dielectric loss because of the molecular relaxation and water polarization that may be present due to ambient conditions such as salinity, temperature, and precipitation [16]. The apparent dielectric permittivity (*K_a_*) is derived from the propagated velocity of an electromagnetic pulse that travels along the TDR sensor probe of a known length [16]. *K_a_* is measured by the sensor as water moves along the soil column. Ref. [18] expressed a method to describe *K_a_*, which included both *ε_r_* and *ε_i_* (Equation (1)):

(1) $$K_{a}=\frac{\varepsilon_{r}}{2}*(1+\sqrt{1+\tan^{2}\frac{\varepsilon_{i}}{\varepsilon_{r}}})$$

where *ε_i_* represents the electric loss factor expressed in terms of either electrical conductance or molecular relaxation [19].

(2) $$\varepsilon_{i}=\varepsilon_{rel}+\frac{\sigma_{DC}}{2\pi f\varepsilon_{v}}$$

where *ε_rel_* is the molecular relaxation, f is the measurement frequency (MHz), *ε_v_* is the permittivity of the vacuum, and *σ_DC_* is the low frequency direct current electrical conductivity. The introduction of soluble salts alters electrical conductivity and increases *ε_i_*. Molecular relaxation is related to the lag in molecular polarization with respect to a changing electric field. When the electric field is oscillated at higher frequencies, the polarization loses energy and the *ε_i_* increases [20]. This effect is more prevalent for higher-salt systems, as there will be an inflated soil moisture measurement because *K_a_* will increase due to the conductivity component of *ε_i_* (Equation (2)).

Many dielectric-based soil moisture sensors, such as TEROS [21] and SOILVUE [22], use *K_a_* to measure soil moisture, while others like the Stevens Hydraprobe II [23] use *ε_r_* to estimate soil moisture. The Stevens Hydraprobe II (Hydraprobe) has the benefit of measuring *ε_r_* and *ε_i_* separately. Separate *ε_r_* and *ε_i_* measurements provide an opportunity to account for the dielectric loss caused due to *ε_i_* sensitivities such as salt concentration and water polarization. Generally, *ε_i_* measurements are often much smaller compared to *ε_r_*. Therefore, with the Hydraprobe sensors, *ε_r_* is considered the dominant factor in *K_a_* readings [9,16] and uncertainties in measurements. With the selected term (*K_a_* [21,22] or *ε_r_* [9,23]), the volumetric soil moisture content (*θ*) is determined through an empirical relationship (Equation (3)):

(3) $$\theta =A\sqrt{K_{a}\;or\;\varepsilon_{r}}+B$$

where *A* and *B* are soil-specific calibration coefficients. For the Hydraprobe, the default A and *B* are 0.109 and −0.178, respectively [23], given a loam soil type. The Stevens Hydraprobe [23] study claims that this default calibration is suitable for all soil types, but recommends the possibility of generating custom calibration if porosity is higher than 60%. The porosity of the site used in this research ranges from 59% to 67%, which is typical of GSI, and as such could benefit from custom calibration.

There are many calibration equations to determine volumetric moisture content based on the measured dielectric properties of soil. [16] introduced a third-degree polynomial relationship between *K_a_* and volumetric water content that provides a 1.3% error of estimates over the entire range of water content tested. Another common calibration procedure is to measure the *K_a_* or *ε_r_* dielectric constant from the sensor and correlate it with the soil moisture content measured through a thermo-gravimetric test for the specific soil sample [8,10,24]. In this process, a set volume of water is added to a defined container of soil equipped with soil moisture sensors, and the water drains out after a desired wet condition is met.

Soil moisture sensors are sensitive to installation; therefore, any air gaps create a variation in actual soil moisture measurement [22,25]. Soil moisture measurements are also subtly impacted by surrounding ambient conditions (e.g., salinity, temperature change, and volume and intensity of water flow). Several studies [8,9,10,12,16] provide analysis of the soil moisture of the subsurface using soil moisture sensors with soil-specific calibrations, but their calibrations are confined to a controlled environment (i.e., a laboratory). In the present study, soil moisture measurements and calibration functions are applied to not only the controlled laboratory environment, but to a GSI field setup with varying installation and ambient conditions that may demonstrate the importance of dielectric losses, especially those of *ε_i_*. The field conditions that are not present in the laboratory (such as the presence of salts) can lead to differences between actual soil moisture and soil moisture sensor measurement. It is necessary to quantify these associated uncertainties to provide useable and interpretable data for stormwater applications. The purpose of the study is to quantify the impact of soil moisture sensor variation on GSI performance on an annual basis, which is discussed through subsurface indicators such as soil moisture recession, desaturation time, and soil moisture measurement at saturation.

## 2. Methods and Materials

There were three main steps in evaluating the soil moisture sensors. In all steps, the Stevens Hydraprobe II was used, which measures *ε_r_* and *ε_i_*, directly and separately, and *K_a_* was calculated using Equation (1). First, the soil moisture sensors were tested using five different media (air, water, DI water, soil slurry, and salt solution) to quantify the *ε_r_*, *ε_i_*, and *K_a_* and associated soil moisture measurements. The purpose of testing sensors in these media was to understand soil moisture measurements at extreme conditions such as air (moisture is 0) and water (moisture is 1), impacts of salt intrusion, and to know whether *ε_i_* had an impact on soil moisture measurements. Second, the sensors were installed in two different laboratory soil setups (one destructive soil setup and one soil box with a defined water flow path) to pursue the following objectives: 1. understand the differences between sensor-measured soil moisture versus the volumetric soil moisture estimated from gravimetric samples, and 2. develop a calibrated empirical relationship between dielectric permittivity and volumetric soil moisture content. Third, an empirical calibration relationship was developed for field-deployed soil moisture sensors and compared against field soil samples.

### 2.1. Verifying Dielectric Constant from Sensors

The dielectric constant values for the soil moisture sensors were tested using tap water, distilled water, soil slurry, salt solution, and air to verify the lower and upper limits of the dielectric readings. In each medium, two soil moisture sensors were tested (labeled Hydraprobe 1 and Hydraprobe 2). Distilled water, tap water, and air media have known values of *K_a_* and *ε_r_* that can be used to verify sensor accuracy, specifically 81.5 for water and 1 for air at a controlled temperature of 20.5 °C [16]. The loamy sand soil slurry consisted of soil and tap water in the ratio of 1:3, and the salt solution consisted of 167 g of added NaCL in 19,255 cm^3^ of tap water. Sensed soil moisture, temperature, real dielectric permittivity, and conductivity were measured for all media.

### 2.2. Laboratory Soil Setup

**Figure 1:**
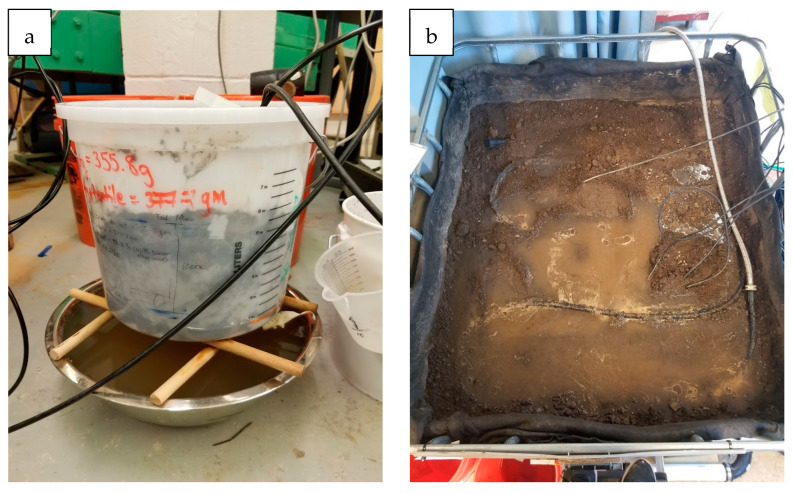
Laboratory setup (**a**) bucket setup (**b**) soil box.

Two soil setups were prepared in the laboratory under controlled temperatures between 20 °C and 25 °C. The first setup was a bucket (23 cm in diameter) with bottom drain holes (Figure 1a) filled with a known mass of loamy sand soil 14 cm deep, with the soil moisture sensor installed at a depth of 10 cm. The primary goal of this test was to compare the volumetric moisture content from the thermogravimetric technique (TGM) and the soil moisture measurement from the default soil moisture sensor. The idea was to understand what sort of variation in soil moisture is produced due to different soil types (loamy sand) from the container versus the factory-general-calibrated (loam) sensor measurements.

The second setup (Figure 1b) was a soil box (91.4 cm by 121.9 cm by 73.2 cm deep) with loamy sand soil. Two soil moisture sensors were installed at 5 cm and 10 cm depths in the center of the box. The box had a fixed inflow at the top of the soil surface and an outlet at the bottom of the soil. The goal of this soil setup was to develop a laboratory-based soil-specific calibration coefficient (A and B from the Equation (3)) for the soil moisture sensors.

A loamy sand soil, typical of GSI media, was chosen for both laboratory soil setups, composed of 79 percent sand, 20 percent silt, and 1 percent clay, according to the USDA classification. The soil was placed in the box and lightly compacted in lifts to be representative of a loosely compacted GSI field site of 1.1–1.2 g cm^−3^. The first soil container setup was compacted to mimic the density in the GSI. The USDA soil classification for the field samples was also loamy sand (the same soil type as the laboratory setups); however, the sand content was higher, ranging between 72 and 85 percent [26].

Using the first setup, 64 soil samples were collected over 33 iterations. Because the soil mass was known, a mass balance was used to calculate the bulk density of the soil from which volumetric moisture content was estimated. The dry density recorded for the 64 samples averaged approximately 1.1 ± 0.06 g cm^−3^. For both setups, the dry density was used to convert the gravimetrically obtained soil moisture samples to volumetric soil moisture to compare with sensor readings.

The first soil setup was destructive, meaning that once the soil samples were collected from an iteration, the soil setup was re-installed. Different physical conditions were established to replicate three important soil conditions: wilting point, field capacity, and saturation. Each scenario was tested 6, 48, and 10 times, respectively. Although these points (wilting point, field capacity, and saturation) are traditionally related to a single suction value on a soil water characteristic curve, other studies define them as a range. For example, field capacity may be within the range of 61–337 cm depending on the texture, structure, and organic content of the soil [27,28]. The goal of this method was to create physical scenarios that enabled the measurement of soil moisture near or around these important points as well as to capture the function range of the soil moisture meter in soil.

The soil sample at saturation was collected when the bucket was filled with water until ponding occurred, and the underdrain holes were just touching the water surface of the catchment below the bucket. The setup was left untouched for a day to ensure that the entire soil was saturated, and the water level was maintained. The field capacity soil samples were collected after the gravitational water drained out. One to three soil samples were collected a few hours after draining, while one to four samples were collected 1–3 days later. To mimic a dry condition to approximate wilting point (note that wilting point cannot be directly obtained, as there are no plants in this system), samples were collected after 3–4 weeks; during this time, water was able to drain freely out of the bottom or evaporate from the soil.

In the second setup, soil samples from the soil box were collected at a depth of 5 cm, adjacent to the location of the 5 cm sensor, using 5 cm long soil cores for different soil moisture conditions ranging from saturation to field capacity. The procedure to obtain saturation and field capacity was similar to the first setup. These soil cores were stored in an air-tight container to prevent moisture loss through evaporation prior to analysis. Altogether, 22 samples were collected, including multiple duplicate samples. The average of duplicated samples was taken, and outliers were removed, of which five data samples were used to calibrate the soil moisture measurement in the laboratory.

### 2.3. Field Soil Sampling

**Figure 2:**
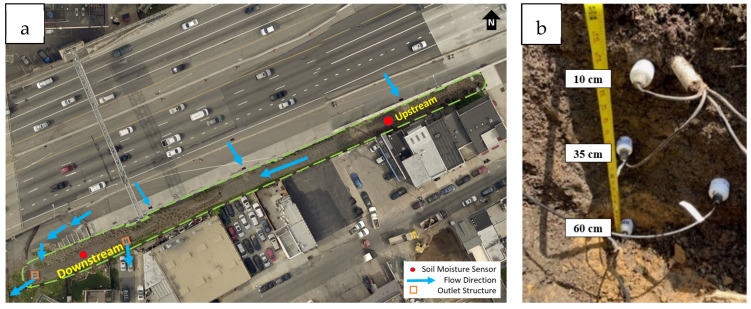
(**a**) Field site of I-95 project at Girard Avenue along with (**b**) location of soil moisture sensors installed at distinct soil depths.

The field site used in this research is a linear bioswale [1] GSI located in Philadelphia, PA (Figure 2a). The GSI collects highway runoff, and there is 60 cm of engineered loamy sand in the bioswale to enhance infiltration. Four Stevens Hydraprobe II [29] soil moisture sensors were each used at two locations (upstream and downstream, Figure 2b) and are located at three vertical depths (10 cm, 35 cm (two sensors at this depth), and 60 cm) below the soil surface. An on-site weather station collects rainfall via a tipping bucket rain gauge [30], and all data is logged in 5-min intervals.

**Figure 3:**
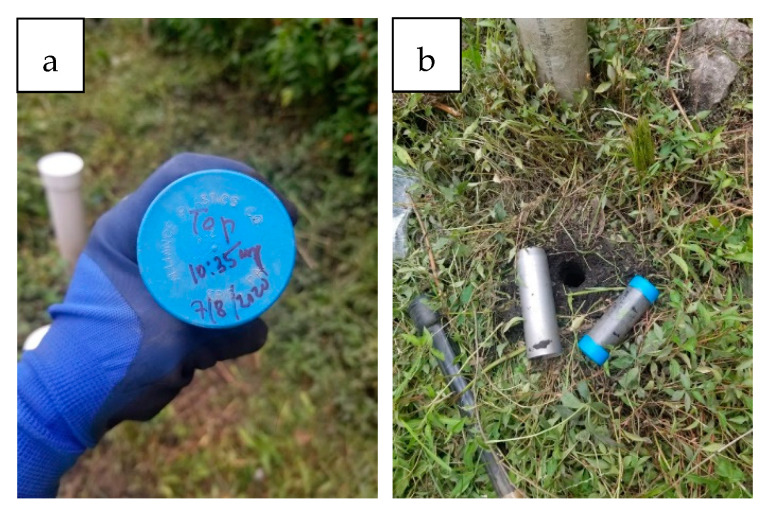
Soil sample collection from the field; (**a**) date and time of the sampling core, (**b**) collected soil core from the field.

A total of 20 (10 per location) soil samples were collected to represent the 10 cm depth reading at both upstream and downstream locations over ten events. The samples were collected using 15 cm long soil cores taken from the surface (after the first few centimeters of highly organic top layer was pushed aside) and were analyzed for volumetric soil moisture with the thermo-gravimetric method. The exact time the soil samples were collected (Figure 3) was noted and referenced with the *ε_r_* and *ε_i_* sensor readings. To mimic existing soil moisture, samples were collected between a few hours after the rain event stopping to 1–2 days after a rainfall event. This replicated the field capacity soil moisture values that ranged from 0.311 m^3^ m^−3^ to 0.350 m^3^ m^−3^. The wilting point was difficult to estimate, as the GSI site had frequent rainfall events captured.

### 2.4. Calibration Process

For the calibration process, the *ε_r_* and *ε_i_* recorded by the sensors were noted for the exact time when the soil samples were collected. To conduct a thermo-gravimetric test, soil samples were placed on a tray to oven dry for 24 h at 110 °C. The gravimetric water content was calculated, which gave the volumetric soil moisture, using a bulk density between 0.9 g cm^−3^ and 1.1 g cm^−3^, as obtained via soil core testing. The estimated volumetric water content (θ) was correlated with the *ε_r_* of the soil sensors using Equation (3). The *K_a_* was calculated using Equation (1) and was calibrated using Equation (3) by considering *K_a_* instead of *ε_r_*. By doing this, variations of soil moisture due to *ε_i_* that are present in other types of soil sensors can be estimated. This calibration process was carried out for the laboratory soil setups and for the field collected sample tests.

### 2.5. Statistical Analysis

To statistically validate the soil moisture measurements obtained from factory general calibration, lab calibration, and the *ε_r_* and *K_a_* associated calibration, a paired *t*-test was performed. The paired *t*-test evaluates whether there is a statistically significant difference between the means of two related datasets. The comparisons were conducted for 1. volumetric soil moisture vs. factory-calibrated soil moisture, 2. volumetric soil moisture vs. lab-calibrated soil moisture, 3. volumetric soil moisture vs. *ε_r_* associated soil moisture, and 4. volumetric soil moisture vs. *K_a_* associated soil moisture. The significance level was set at *p* < 0.05.

## 3. Results and Discussions

### 3.1. Verifying Dielectric Constants

The purpose of verifying the dielectric constant is to check the readings of sensors in different environmental scenarios and to learn the impacts of *ε_i_*. The range of the dielectric constant recorded in the five selected media is about 1 in air to about 81 in water, which is similar to what Topp et al. (1980) specified: 81.5 for water and 1 for air at controlled temperature of 20.5 °C; Table 1). Tap water and distilled water were expected to provide a moisture content measurement of 100 percent; however, the sensor never measured over 80 percent, which indicates a limitation in the sensor. For pure air and water conditions (where soil moisture is 0 or 100 percent, respectively), which are unlikely to be experienced in field conditions, the variations in soil moisture (*θ*) associated with *K_a_* and *ε_r_* were negligible between each sensor (Table 1; Hydraprobe 1 and 2). A relatively small (<5%) percent difference was observed between the soil moisture determined by *K_a_* (*θ*[*K_a_*]) and by *ε_r_* (*θ*[*ε_r_*]) for each sensor for the soil slurry, and for one sensor for the salt solution, while a larger percent difference (26%) in *θ* was observed for one salt solution sample. According to the Stevens Hydraprobe [29], the sensor precision ranged from ±0.01 to ±0.03, which was seen in the soil slurry samples, but not the salt solution. This observation demonstrates the sensitivity of *ε_i_* with soil texture and soluble salt concentration, which negates the assumption that *K_a_* can be approximated as *ε_r_* [16]. To further validate this finding, soil moisture measurements along with dielectric permittivity were studied in the laboratory and field soil setups.

### 3.2. Soil Sampling Analysis—Laboratory Setups

**Figure 4:**
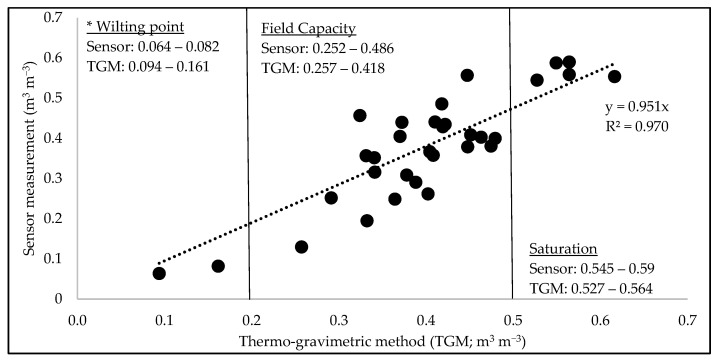
Volumetric moisture content comparison of sensor and soil samples using first bucket setup. * The wilting point was determined based upon the dry soil after an extended “drought” period.

The volumetric soil moisture measurement from the thermo-gravimetric method (TGM) was compared to the sensor-recorded moisture data from the first laboratory setup (bucket setup; Figure 4). Altogether, 64 samples were taken, and for some sensor readings, multiple soil samples were collected and duplicated. The factory default sensor measured soil moisture as slightly less than that of the thermo-gravimetric estimated volumetric soil moisture (R^2^ = 97%) when intercepted through the axes. The variation in soil moisture measurement between the sensor and thermo-gravimetric methods ranged from ±0.005 to 0.14 (Figure 4).

The samples taken to represent wilting point ranged from 0.06 to 0.08 m^3^ m^−3^ in the sensor but 0.09 to 0.16 m^3^ m^−3^ by established laboratory methods; as such, these ranges, though close, did not overlap. The samples taken to represent field capacity ranged from 0.25 to 0.49 m^3^ m^−3^ in the sensor and 0.25 to 0.42 m^3^ m^−3^ by established laboratory methods and had a very similar range to one another. Finally, the samples taken to represent saturation ranged from 0.55 to 0.59 m^3^ m^−3^ in the sensor and 0.53 to 0.56 m^3^ m^−3^ by established laboratory methods and had a similar range, with an overall higher range for the sensor. Overall, the factory general calibration seems to underestimate lower values of soil moisture and overestimate higher values of soil moisture. The factory default calibration of “loam” claims to be applicable to almost every mineral soil [9], but the known soil type is loamy sand, which could be partly attributable to the difference between the sensor and the thermo-gravimetrically found values.

**Figure 5:**
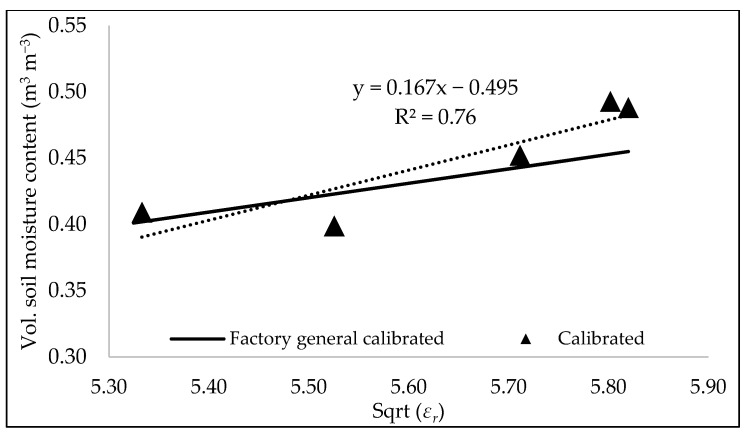
The measured relationship between the dielectric constant (*ε_r_*) and volume soil moisture content at the laboratory setup.

In the second soil setup (soil box), 22 samples were collected. Based on these samples, the calibration coefficients (Equation (3)) for loamy sand were determined from the regression equation, as A is 0.167 and B is −0.495 (Figure 5), with an R^2^ value of 0.76. These values are different from the manufacturer’s default recommendations (i.e., a loam soil type) of A and B as 0.109 and −0.178, respectively. The dry density of 1.1 g cm^−3^ of the soil samples collected was mimicked in the lab. Differences in density between the manufacturer’s default calibration (loam calibration) and the lab-determined calibration coefficients are assumed to be the driving reason for the difference. A major limitation of the soil box experiment was the number of samples used for estimating calibration coefficients. The typical wetting and drying cycle experienced in the field that results in large variations in soil moisture was difficult to obtain in a large soil box laboratory setting without plants and sun exposure, such that soil moisture values hovered mostly between 0.4 and 0.5 m^3^ m^−3^. These limitations prevented any conclusion as to whether the laboratory calibration was an improvement over the manufacturer curve. As the primary goal of this research was to quantify uncertainties due to environmental conditions, the focus was shifted towards using both the default (called factory general calibration) and lab-determined calibration such that each calibration could be compared to what is observed in the field.

### 3.3. Comparing Laboratory Coefficients with Factory General Coefficients

**Figure 6:**
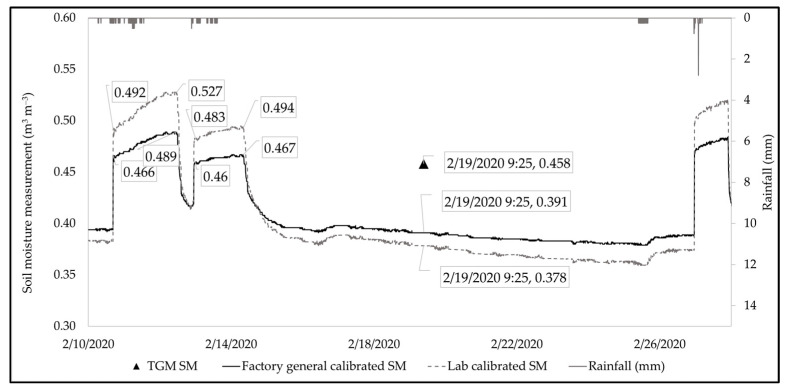
Comparison of soil moisture data from factory-general-calibrated sensors, laboratory-calibrated equation, and soil moisture calculated from thermo-gravimetric method.

The laboratory-calibrated coefficients were applied to soil moisture sensors installed at the field site to understand variations in soil moisture in the field condition. The soil moisture profile from a series of storm events at the field site, at a 10 cm depth, is presented in Figure 6. The dotted line represents the laboratory-calibrated soil moisture profile (A = 0.167, B = −0.495) and the solid line is the factory-general-calibrated soil moisture profile (A = 0.109, B = −0.178). The saturated soil moisture (which is the soil moisture after the abrupt rise until it starts to recede) for the factory-general-calibrated profile and laboratory-calibrated profile ranges from 0.46 m^3^ m^−3^ to 0.489 m^3^ m^−3^ and 0.492 m^3^ m^−3^ to 0.544 m^3^ m^−3^, respectively. A paired *t*-test showed a statistical difference between the two datasets (factory-general-calibrated and laboratory-calibrated). Some differences between datasets could be due to differences in soil type and/or compaction level, but they also could be influenced by the presence of accumulated organics at the field site since its construction in 2015.

A grab sample was collected from the field site on 19 February 2020 at 9:25 am (triangle in Figure 6). The volumetric moisture content for the collected grab sample was 0.458 m^3^ m^−3^ (triangle in Figure 6), which falls at the bottom of the range for the saturated condition of the factory-general-calibrated sensor readings, although it was lower than the range for the calibrated sensor readings. Because the soil sample from the field was collected several days after the rain event occurred, theoretically the soil moisture would have been at its field capacity, i.e., the moisture value should have been less than the soil moisture at saturation; however, the grab sample value (i.e., 0.458) was higher than the soil moisture sensor measurement (Figure 6; soil moisture ~0.378). This observation demonstrates uncertainty regarding the use of laboratory-based soil-specific calibrations for field applications. Having more data samples would provide an opportunity to compare the manufacturer-based and laboratory-based calibration to determine if laboratory measurement is superior to the manufacturer’s calibration. However, field-based calibration was pursued in hopes that it would yield more certainty in in situ soil moisture readings and ongoing climatic conditions, also considering the moisture condition of the actual GSI soil.

### 3.4. Field Calibration

The field sensors calculate soil moisture based on *ε_r_*, but to identify any variation in soil moisture due to *ε_i_*, the soil moisture was also calculated using *K_a_* (Equation (1)). Comparing the sensor readings to the volumetric soil moisture measurement of the 20 soil samples, Equation (3) was used to find field calibration coefficients for the soil moisture sensor. The value ranged from 0.311 m^3^ m^−3^ to 0.350 m^3^ m^−3^. This range of data agreed with the field capacity range estimated through the traditional SWCC curve, which is 0.33 m^3^ m^−3^ to 0.47 m^3^ m^−3^ [1].

**Figure 7:**
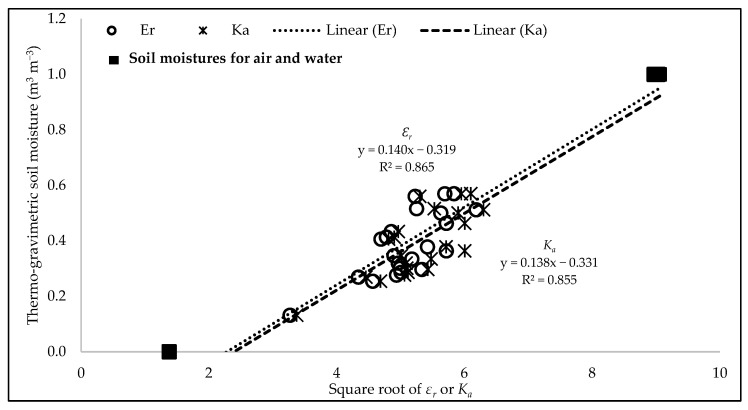
Field calibration of soil moisture sensors at field GSI site.

In Figure 7, the circle and asterisk represent the soil moisture calibration for the field site soil (i.e., loamy sand) with *ε_r_* and *K_a_*, respectively. In addition to the volumetric soil moisture from the gravimetric samples, the values for air and water from Table 1 (solid square dot) were also added in the graph to show the absolute minimum and maximum limits. A paired *t*-test showed no statistical difference between the two datasets (*ε_r_* and *K_a_*). However, the field-calibrated coefficients associated with *ε_r_* are A: *ε_r_* = 0.140 and B: *ε_r_* = −0.319; while those associated with *K_a_* are slightly different as A: *K_a_* = 0.138 and B: *K_a_* = −0.331.

**Figure 8:**
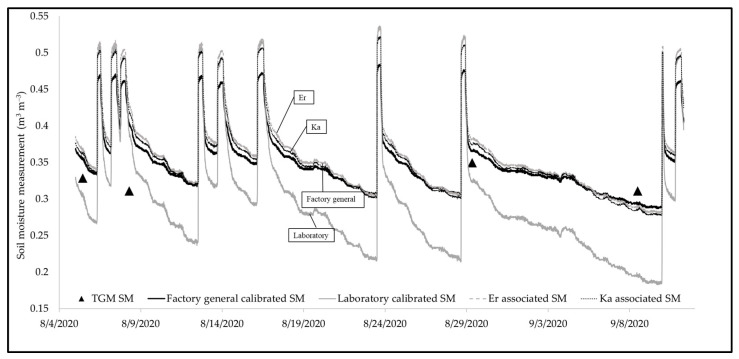
Comparison of sensor-recorded soil moisture (SM) data with uncalibrated, laboratory-calibrated and field-calibrated soil moisture associated with *εr* and *K_a_*, along with volumetric soil moisture calculated through thermogravimetric method (TGM).

Figure 8 contains an example of the soil moisture profiles for one field site location with the following elements: (1) factory-general-calibrated equation (solid black; A = 0.109, B = −0.178), (2) laboratory-calibrated equation (solid grey; A = 0.196, B = −0.654), (3) field-calibrated equation using *εr* (dashed; A = 0.140, B = −0.319), and (4) field-calibrated equation using *K_a_* (dotted; A = 0.138, B = −0.331). The triangular points represent grab sample soil moisture measurements. The soil moisture profile generated from the laboratory calibration varies distinctively from the rest. The lowest soil moisture value for a laboratory-calibrated sensor reads 0.183 m^3^ m^−3^, which is below the soil moisture at field capacity, according to the bucket test (Figure 4). The recession according to the laboratory-calibrated sensors is abrupt compared to the other three soil moisture profiles, as it varies more than others when compared to the soil moisture estimated through the thermo-gravimetric method. In contrast, the field-calibrated soil moisture measurements are closer to the factory-general-calibrated soil moisture measurements and within a reasonable range of the grab soil samples. This observation demonstrates that the laboratory calibration does not fully capture the reality of the field subsurface layer and indicates that the soil moisture from field-calibrated sensors is likely more dependable than the laboratory-based calibrations.

Among the three soil moisture profiles—(1) factory-general-calibrated (solid black), (2) field-calibrated with *ε_r_* (lighter dashed grey), and (3) field-calibrated with *K_a_* (darker dashed grey) sensors (Figure 8)—variations in soil moisture measurements were more noticeable at the saturation value compared to field capacity. The average maximum soil moisture at saturation for profile 1, 2, and 3 were about 0.47 m^3^ m^−3^, 0.53 m^3^ m^−3^ and 0.5 m^3^ m^−3^, respectively. The variation in soil moisture measurement at saturation between the factory-general-calibrated sensors and the calibrated sensors was about 6% and 3% with *ε_r_* and *K_a_*, respectively. The calibration equation associated with *K_a_* was selected for quantifying the variations associated with the soil moisture sensors. The reasoning for this is, firstly, that soil moisture sensors generally can only quantify *K_a_*, and it is of interest to be able to understand the variability between using a more costly sensor that can measure *ε_r_* and a more common sensor that can measure *K_a_* in a field system. In this case, it happened that measurements for saturation with *K_a_* were closer to the derived equations that estimate soil moisture measurements [31], unlike in a previous study [16]; neglecting *ε_i_* provided an error of estimates of 1.3% for the studied range of soil moisture. In the present experiment, the resulting *ε_r_* and *K_a_* calibrations were not very different (Figure 8). The largest difference between the *ε_r_* and *K_a_* calibration was around saturation (a 6% difference in calibration was found associated with *ε_r_*, and a 3% difference was found associated with *K_a_* (which considers *ε_i_*) for saturation)). It should be noted that there was no snow on this GSI during this study; therefore, effects associated with salinity did not play a large role in the soil sensor measurements. Therefore, in this case, it was found that the calibration-associated *K_a_* could be used to establish the empirical relationship between *K_a_* and the soil moisture measurements.

### 3.5. Statistical Significance

The results of paired *t*-tests are summarized in Table 2. The *p*-values for all comparisons are greater than 0.05, indicating no statistically significant differences between the volumetric soil moisture and the factory-general-calibrated, lab-calibrated, or *ε_r_*- and *K_a_*-associated soil moisture values.

The results of the paired *t*-tests demonstrate no statistically significant differences (*p* > 0.05) between volumetric soil moisture (TGM SM) and soil moisture values obtained through factory general calibration, lab calibration, and *ε_r_*- and *K_a_*-associated soil moisture measurements. This indicates strong consistency across calibration methods, confirming the reliability of sensor-based measurements for estimating soil moisture. The observed variations are likely due to measurement uncertainties rather than systematic errors, which is expected due to environmental factors, sensor resolution, and sample variability. This validation is particularly important for monitoring GSI performance, as consistent and reliable soil moisture data are critical for evaluating subsurface hydrologic behavior. The findings suggest that calibrated sensor measurements can serve as a practical and cost-effective alternative to gravimetric sampling, reducing the burden of frequent manual measurements. Future work could further quantify uncertainties using advanced statistical methods to improve the precision and interpretation of soil moisture data.

### 3.6. Implementing Calibration Associated with K_a_ to Understand Soil Moisture Variation

A study by Shakya et al. [1] provides a conceptual framework of a typical soil moisture profile (ABCDEF” F) along with the time at the end of ponding (P_e_). Notations ABCDEF are the soil moisture measurements that represent the pre-storm condition, beginning of saturation, end of saturation, field capacities (D and E) and post-storm conditions, respectively. The time duration from C to F” is the soil moisture recession that can be used to evaluate the recovery of a GSI system for a given rainfall event.

**Figure 9:**
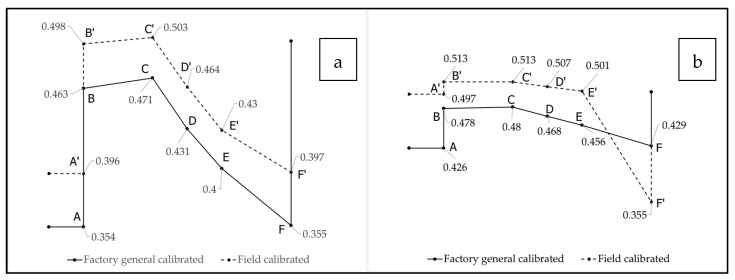
Average soil moisture measurement at GSI for entire studied event at distinct soil depths of (**a**) 10 cm (**b**) 35 cm.

Average soil moisture measurements were calculated using the field-calibrated soil moisture, using *K_a_* for 55 rainfall events that occurred from September 2019 to October 2020 (Figure 9). For both the 10 cm and 35 cm soil moisture sensors, on average, there was about a 3% change in soil moisture measurements at saturation and field capacity. The pre-storm (point A) and post-storm (point F) soil moisture conditions are dependent on the antecedent dry time and weather conditions during the dry time (e.g., temperature, humidity, and wind); therefore, the soil moisture variations are slightly different when comparing saturation (point B and C) and field capacity (point D and E).

**Figure 10:**
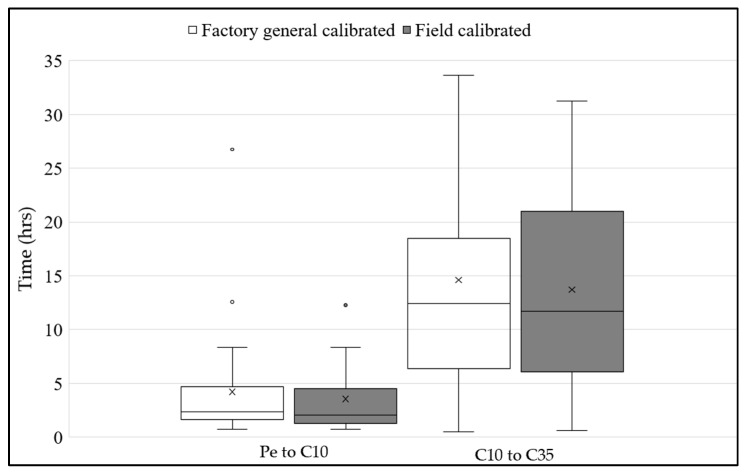
Time variation in water movement along the soil column between factory-general-calibrated and calibrated soil moisture.

The time taken by water (desaturation time) to move from the surface to the 35 cm soil depth was noted at three different depths: 0 cm at the end of ponding (Pe), C at 10 cm (C10), and C at 35 cm (C35). In Figure 10, the average desaturation times for water to move from the Pe to C10 and C10 to C35 for the factory-general-calibrated sensors are 4.2 h and 14.6 h, respectively, while for the calibrated (using *K_a_* field calibration) sensor, the average desaturation times are 3 h and 13.7 h, respectively. According to the calibrated sensors, the water desaturation time was faster by 1 h.

**Figure 11:**
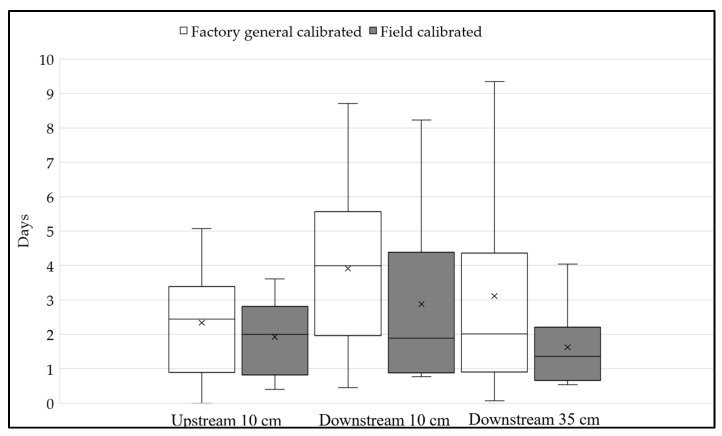
Variation in soil moisture recession between factory-general-calibrated and calibrated soil moisture profiles.

Soil moisture recession (the time for soil moisture to reach from point C to point F”) was estimated for the upstream 10 cm, downstream 10 cm, and 35 cm soil moisture sensors to quantify GSI system recovery (Figure 11). On average, the soil moisture recession time for upstream 10 cm, downstream 10 cm, and 35 cm are 2.5 days, 3.9 days, and 3.1 days, respectively, with the factory-general-calibrated sensor. With the calibrated sensor, the recession time decreased to 1.9 days, 2.9 days, and 1.6 days at upstream 10 cm, downstream 10 cm, and 35 cm, respectively. Overall, the factory-general-calibrated sensors indicate it takes 3–4 days for the GSI system to recover to pre-storm conditions, while the calibrated sensors indicate 2–3 days.

**Figure 12:**
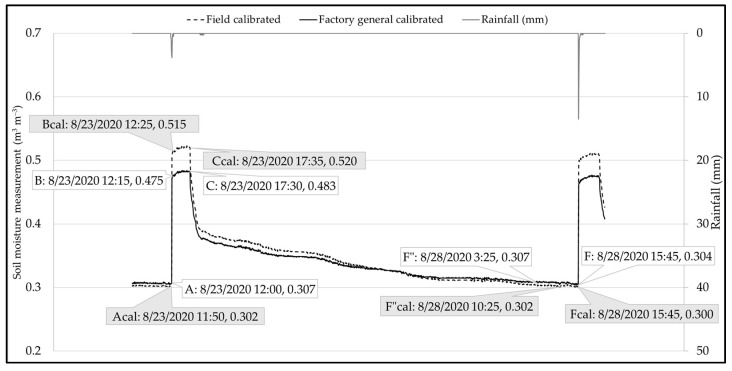
Example rainfall event presenting the variation in GSI recovery between factory-general-calibrated and field-calibrated soil moisture profiles.

An individual rainfall event was selected for an in-depth analysis of recovery time. In Figure 12, there was a 7 h difference in recession time between the factory-general-calibrated and field-calibrated sensor. While the soil moisture measurements at pre-storm, post-storm, saturation, and field capacity remain consistent, the differences observed between the field-calibrated and factory-general-calibrated sensors do have an impact on system recovery time. This is an essential consideration from a system design perspective, as reducing the recession even by a few hours or full day could affect the water storage capacity of a GSI system in the long run.

## 4. Conclusions

In this study, variations associated with soil moisture sensors to predict moisture conditions for different flow conditions were explored. It was found that there is a variation in soil moisture measurement when the sensors are calibrated with soil-specific coefficients versus the default or factory-general-calibrated coefficients. The calibration was conducted in both a field and lab environment, which included an empirical relationship between dielectric properties (*ε_r_*, *ε_i_*, and *K_a_*) and volumetric soil moisture. The difference in soil moisture with calibration in the laboratory soil setup is minimized, but there are still doubts around the applicability of the measurements to the field. To ultimately represent the in-situ conditions, calibration in an uncontrolled environment (or rather, the field setup where the soil moisture sensors will ultimately be installed) is preferrable, as it produced less variation in soil moisture measurements. The calibrations were discussed through the importance of both *ε_r_* and *K_a_*. *K_a_* was ultimately used, as it did not result in a very different calibration than *ε_r_*, and both *ε_r_* and *K_a_* field calibrations were able to align well with in situ changes in the GSI system, which is generally not included in the laboratory-based calibration methods studied so far. Statistical validation through paired *t*-tests further confirmed no significant differences (*p* > 0.05) between volumetric soil moisture and *ε_r_*- and *K_a_*-associated soil moisture, reinforcing the reliability of these calibrations for field applications. In the future, we encourage exploring the sensitivity of *ε_i_* in the calibration process, as *ε_i_* is directly affected by changes in water polarization, which was likely not a large factor in the study time frame.

Overall, this improved understanding of variation in soil moisture sensors can further our understanding of GSI hydrology. Depending on the sensor or the calibration (i.e., default coefficients or field-calibrated), there will be some differences in observations, such as the time of soil moisture recession after an event, which indicates a GSI’s readiness for the next storm event. Recognizing that there is some uncertainty can help designers test a variety of design scenarios to see how significant changing a design element, such as media type or drainage structure, will be for GSI performance. However, this study faced several limitations. The scope of field and laboratory data collection was restricted in duration, potentially overlooking broader seasonal and long-term variations in soil moisture dynamics. Additionally, the study did not evaluate the effects of salt intrusion, a factor that can significantly influence the dielectric properties of soil, especially *ε_i_,* and, consequently, sensor accuracy. Furthermore, the research was limited to a single study location, which constrains the generalizability of its findings to other GSI systems with differing soil and environmental characteristics. Despite the limitations, this research demonstrated how soil moisture sensors can play a role in identifying the expected range of performance of a GSI system. As GSIs are dynamic systems that have seasonal and life-time variations, it is useful to know how soil moisture during and after a storm contributes to that variation. With this variation, a better understanding of expected performance and associated maintenance needs can be made.

## Figures and Tables

**Table 1:** Raw sensor measurements for soil moisture and dielectric permittivity.

Media	Sensors	*ε* *_r_*	*ε* *_i_*	*K_a_* ^a^	*θ*[*ε_r_*] (m^3^ m^−3^)	*θ*[*K_a_*] (m^3^ m^−3^)
Tap water	Hydraprobe 1	80.5	19.3	81.7	0.799	0.807
	Hydraprobe 2	81.6	13.0	82.1	0.806	0.809
Soil Slurry	Hydraprobe 1	67.5	27.3	70.4	0.712	0.737
	Hydraprobe 2	55.9	27.3	59.6	0.636	0.664
DI water	Hydraprobe 1	81.6	1.3	81.6	0.806	0.807
	Hydraprobe 2	81.9	−3.6	81.9	0.807	0.808
Salt Solution	Hydraprobe 1	154.9	113.9	181.9	0.950	1.292
	Hydraprobe 2	193.1	97.0	206.7	1.333	1.389
Air	Hydraprobe 1	1.9	0.3	1.9	0	−0.027
	Hydraprobe 2	1.7	0.0	1.7	0	−0.037

Note: ^a^ The Hydraprobe measured *ε_r_* and *ε_i_* through sensors; *K_a_* values for the Hydraprobe were calculated using Equation (3) (A = 0.109, B = −0.178).

**Table 2:** Paired *t*-test for soil moisture measurements between volumetric soil moisture and factory-general-calibrated, lab-calibrated, and *ε_r_*- and *K_a_*-associated soil moisture.

Comparison	*t*-Statistic	*p*-Value	Significance
Volumetric vs. factory	0.539	0.596	Not significant
Volumetric vs. lab	1.479	0.155	Not significant
Volumetric vs. *ε_r_*	−0.547	0.591	Not significant
Volumetric vs. *K_a_*	−0.715	0.483	Not significant

## Data Availability

All raw soil moisture data that support the findings of this study are available from the corresponding author upon reasonable request.
